# EARN: an ensemble machine learning algorithm to predict driver genes in metastatic breast cancer

**DOI:** 10.1186/s12920-021-00974-3

**Published:** 2021-05-07

**Authors:** Leila Mirsadeghi, Reza Haji Hosseini, Ali Mohammad Banaei-Moghaddam, Kaveh Kavousi

**Affiliations:** 1 Department of Biology, Faculty of Science, Payame Noor University, Tehran, Iran; 2 Laboratory of Genomics and Epigenomics (LGE), Department of Biochemistry, Institute of Biochemistry and Biophysics (IBB), University of Tehran, Tehran, Iran; 3 Laboratory of Complex Biological Systems and Bioinformatics (CBB), Department of Bioinformatics, Institute of Biochemistry and Biophysics (IBB), University of Tehran, Tehran, Iran

**Keywords:** Metastasis breast tumor, Mutation data, Ensemble classifier, Plausible drivers, Targeted clinical panel sequencing

## Abstract

**Background:**

Today, there are a lot of markers on the prognosis and diagnosis of complex diseases such as primary breast cancer. However, our understanding of the drivers that influence cancer aggression is limited.

**Methods:**

In this work, we study somatic mutation data consists of 450 metastatic breast tumor samples from cBio Cancer Genomics Portal. We use four software tools to extract features from this data. Then, an ensemble classifier (EC) learning algorithm called EARN (Ensemble of Artificial Neural Network, Random Forest, and non-linear Support Vector Machine) is proposed to evaluate plausible driver genes for metastatic breast cancer (MBCA). The decision-making strategy for the proposed ensemble machine is based on the aggregation of the predicted scores obtained from individual learning classifiers to be prioritized homo sapiens genes annotated as protein-coding from NCBI.

**Results:**

This study is an attempt to focus on the findings in several aspects of MBCA prognosis and diagnosis. First, drivers and passengers predicted by SVM, ANN, RF, and EARN are introduced. Second, biological inferences of predictions are discussed based on gene set enrichment analysis. Third, statistical validation and comparison of all learning methods are performed by some evaluation metrics. Finally, the pathway enrichment analysis (PEA) using ReactomeFIVIz tool (*FDR* < 0.03) for the top 100 genes predicted by EARN leads us to propose a new gene set panel for MBCA. It includes HDAC3, ABAT, GRIN1, PLCB1, and KPNA2 as well as NCOR1, TBL1XR1, SIRT4, KRAS, CACNA1E, PRKCG, GPS2, SIN3A, ACTB, KDM6B, and PRMT1. Furthermore, we compare results for MBCA to other outputs regarding 983 primary tumor samples of breast invasive carcinoma (BRCA) obtained from the Cancer Genome Atlas (TCGA). The comparison between outputs shows that ROC-AUC reaches 99.24% using EARN for MBCA and 99.79% for BRCA. This statistical result is better than three individual classifiers in each case.

**Conclusions:**

This research using an integrative approach assists precision oncologists to design compact targeted panels that eliminate the need for whole-genome/exome sequencing. The schematic representation of the proposed model is presented as the Graphic abstract.

**Graphic abstract:**

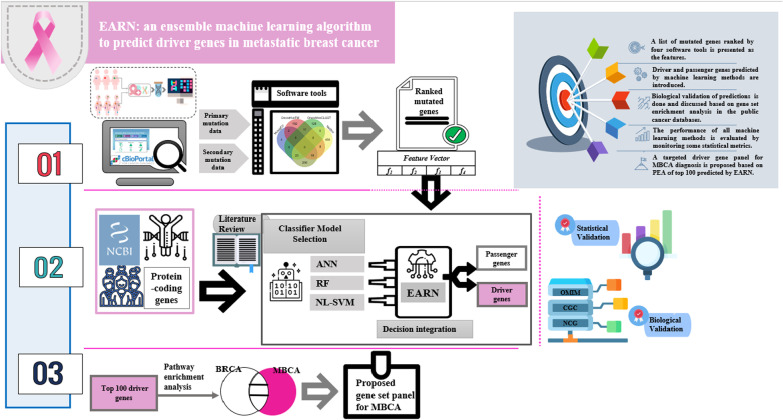

**Supplementary Information:**

The online version contains supplementary material available at 10.1186/s12920-021-00974-3.

## Background

The mutations induce small changes to the genes. If they cause damage and remain untreated, it drives multifactorial anomalies which are called complex diseases. Cancers are one kind of these complex diseases which are induced by defective driver genes and can cause malignant transformation [1]. Among cancers, primary breast cancer as a complex disease is the most commonly diagnosed carcinoma in women worldwide and will be fatal if it progresses towards the secondary-stage. It is the most common and the second most common cause of cancer death in women in developing regions and developed regions, respectively [2]. Over the last 10 years, the incidence of breast cancer has increased almost 10 times [3]. The concern about this growing trend has prompted oncologists to seek early detection. Nowadays, the molecular technique of next-generation sequencing (NGS), including whole-exome sequencing could generate a large amount of data related to mutated genes called mutation data [4]. The analysis of this massive data requires the use of robust computational approaches to exploit the information effectively. Precision oncology by focusing on targeted clinical panel sequencing can be helpful in new treatment targets [5], i.e., a breast cancer-specific NGS panel, including 79 genes has been validated to use in identifying primary and metastatic breast cancer [6]. In this way, the advent of bioinformatics tools in parallel with the development of molecular techniques could lead to discovering biomarkers that are efficient in cancer diagnosis and prognosis [7]. The machine learning algorithms as one of the computational approaches can be trained with data from countless patients whereas it is too difficult for human physicians and biologists to gain such experience in an entire career or their researches. These models equip experts to make better decisions [8]. Some of them are the ensemble classifier (EC) machine learning methods that combine two or several models to optimize the performance of the base components in order to improve data analysis. In previous studies, it has been mentioned that committee approaches can outperform even powerful individual models in many cases [9]. Investigations show using ensemble models (a.k.a fusion systems) is widely increasing in many fields of inquiry, including the detection of cancers and their subtypes and especially in the area of breast cancer detection. In medical researches, ECs have accurately succeeded to improve patients' diagnosis [10]. In 1996, a breast cancer dataset, including 699 samples, were analyzed by bagging nearest neighbor classifiers as a fusion system [11]. Since then, many ensemble classification methods have been applied to breast cancer prognosis [12]. In this regard, we reviewed 42 ensemble methods related to 18 cancers [13]. Among these, 22 approaches have been reported for analyzing breast cancer data in the literature (Table 1).

**Table 1:** 22 ensemble learning methods concerned with the detection of breast cancer

	Method name	Publication year
1	Bayesian networks-based model integration [14, 15]	2006 and 2019
2	RSS-SCS method [16]	2016
3	Collective approach (correlation, color palette, color proportion, and SVM) [17]	2016
4	Kernel-based Data Fusion Method for Gene Prioritization [18]	2015
5	DECORATE method^a^ [19]	2015
6	HyDRA method^a^ [20]	2015
7	GenEnsemble method^a^ (NBS-IB3-SVM-C4.5 DT) [21]	2014
8	NB (Naïve Bayes) combiner method [22]	2014
9	Evolutionary Ensemble Model [23]	2014
10	smoothed t-statistic SVM (stSVM) [24]	2013
11	SVM Classifiers Fusion (three SVM) [25]	2013
12	COMBINER (Core Module Biomarker Identification)^a^ [26]	2012
13	Ensembles of BioHEL Rule Set [27]	2012
14	Stacking IB3-NBS-RF-SVM method [28]	2012
15	REIS-based ensemble method [29]	2011
16	MRS method [30]	2010
17	Boosting-TWSVM method [31]	2009
18	Bagging and boosting-based TWSVM [32]	2009
19	Feature Subsets Method [33]	2008
20	BNCE method [34]	2007
21	Bayesian Network Classifier [35]	2006
22	enSVM (200 SVM) [36]	2006

^a^Some methods that are proposed to discover genomic markers related to breast cancer

In some of these studies, ECs have been used for introducing driver genes associated with breast cancer and the evaluation of genomic biomarkers regarding this cancer. However, little attention has been paid to compare drivers of primary and metastatic tumors in an analytical framework. In this work, we propose the EC learning approach called EARN (Ensemble of Artificial Neural Network, Random Forest, and non-linear Support Vector Machine). It is used to find candidate drivers in primary breast invasive carcinoma (BRCA) and metastatic breast cancer (MBCA) samples from mutation data available in the Cancer Genome Atlas (TCGA) (https://portal.gdc.cancer.gov) and cBioPortal (http://cbioportal.org). The candidate genes introduced by the EC mechanism may already be known as cancers causing genes in databases or can be novel. The candidate genes have the potential to be presented as genomic risk biomarkers after completing the steps of clinical trials [37] and used for personalized targeted therapy [38]. Furthermore, there is evidence that driver genes that effectively prognose cancers could be used in therapeutic applications to access more effective therapies [39]. The proposed EC method combines decisions of three base classifiers, including non-linear Support Vector Machine (NLSVM) [40], Artificial Neural Network (ANN) [41], and Random Forest (RF) [42]. The features for these three classifiers were extracted from four software tools: MutSigCV v.1.4 [43], OncodriveCLUST 0.4.1 [44], OncodriveFM [45], and NetBox 1.0 [46]. Overall, we aim to focus on the findings in five steps of BRCA and MBCA prognosis and diagnosis. 1. A list of mutated genes ranked by four software tools based on *p* value is presented as the features. 2. Driver and passenger genes predicted by three individual machine learning methods and EARN are introduced and compared. 3. Biological validation of predictions based on gene set enrichment analysis is done and discussed. Indeed, we evaluate the top genes predicted by EARN and three base classifiers for BRCA and MBCA by searching these genes in the list of cancer-associated genes in the public databases, including the Online Mendelian Inheritance in Man (OMIM) (http://www.omim.org/), the Cancer Gene Census (CGC) [47], the Network of Cancer Genes (NCG) [48, 49], and the human cancer metastasis database (HCMDB) [50]. 4. The performance of all machine learning methods is evaluated by monitoring some statistical metrics. 5. Finally, a targeted driver gene panel for MBCA diagnosis based on pathway enrichment analysis (PEA) of top 100 predicted by EARN (EARN_100_) is proposed.

## Methods

**Fig. 1:**
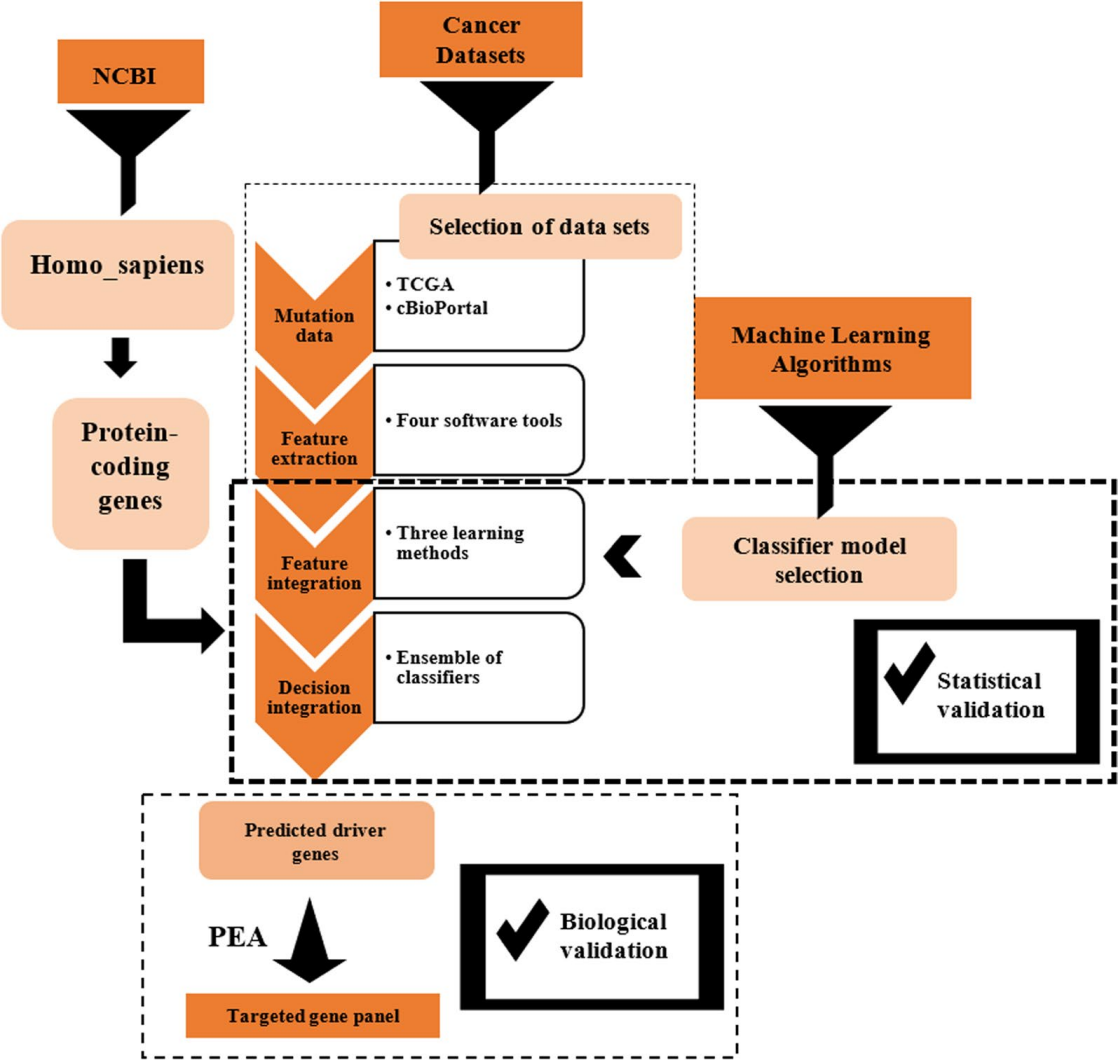
The proposed fusion system workflow for prediction of driver genes in cancers

In this study, an ensemble method as a synergistic combination of computational tools has been designed and proposed to find the putative cancer drivers. This fusion system can help to analyze the Whole-Exome Sequencing (WES) data. It consists of four steps: selection of dataset, feature extraction, feature integration, and decision integration (Fig. 1). We have also shared Python source code and other requirements for the implementation of the proposed ensemble machine learning algorithm as the protocol via GitHub (https://github.com/lmirsadeghi/EARN/).

### Selection of dataset

In this study, to identify candidate driver genes based on mutations that occur in genes, breast cancer primary and metastasis data have been analyzed.

For primary breast cancer, an open-access mutation annotation format (.maf) file was downloaded from TCGA data set regarding BRCA [51]. This file includes 90,969 masked somatic mutations identified in 17,990 genes from 983 tumor samples of BRCA patients that their whole exome had been sequenced by Illumina Genome Analyzer II [see this mutation file in Additional file 1: Table S1]. Also, for processing of sequences, a bioinformatics pipeline framework called "MuSE Variant Aggregation and Masking" in TCGA has been used. For MBCA, two files (.txt) were downloaded from the cBio Cancer Genomics Portal [52, 53]. The first mutation file includes WES of 213 tumor samples from 213 MBCA patients by Illumina HiSeq. It is associated with 22,949 somatic mutation counts that occurred among 10,791 genes [54]. The second file consists of WES of 237 metastasis tumor samples by Illumina GAIIx from 180 patients regarding 24,027 somatic mutations identified in 10,273 genes [55]. Clinical data shows that 86 samples were taken when patients were in the metastatic disease stage, and other samples had been taken less than 4 months prior to the metastatic disease is diagnosed [56]. After selecting two initial datasets concerning MBCA [see these mutation files in Additional file 2: Table S2 and S3], they augmented to build a comprehensive mutation data file, including 46,928 somatic mutations identified among 14,293 genes from 450 MBCA tumor samples (393 patients).

### Selection of software tools for feature extraction

After preparing mutation files, four software tools including MutSigCV v.1.4, OncodriveCLUST 0.4.1, OncodriveFM, and NetBox 1.0 were used to extract the convenient numerical features. The selection of tools for feature extraction was a crucial step to achieve better performance on the final algorithm of the proposed ensemble learning model. We select the four software tools based on evidences of a paper in 2015 on identification and ranking of plausible drivers for BRCA and ovarian (OV) cancer [19]. It had been demonstrated that among ten tools for extracting features, OncodriveFM and NetBox generate high sensitivity, especially about BRCA. Also, the sensitivity of OncodriveCLUST tool is high concerning the OV cancer. On the other hand, in both cancers, it had been shown that the positive predictive value (PPV) for NetBox and OncodriveFM is high. MutSigCV was able to propose a large number of drivers in the top 50 genes for OV, where at least five other methods had also predicted them as top genes. These advantages led us to use these tools. Practically, these four tools evaluate original mutation files from different aspects and assign a score (*p* value) to genes to show their relevance to disease according to that software’s logic. MutSigCV gets data concerning point mutations and small insertions and deletions (INDELs) from the WES file. After analyzing and estimating mutation frequency, it can identify and introduce a significant list of mutated genes for cancers [43]. OncodriveClust software tool is able to identify mutations that generate oncogenes and leads to changes in the function of the proteins. For this purpose, it analyzes synonymous mutations and protein-affecting mutations, including non-synonymous, stop, and splice-site mutations [44]. Also, this tool uses data from the Cancer Gene Census (CGC) database [47]. for selecting known drivers associated with cancers. OncodriveFM is our next tool which can detect driver genes across tumor samples, identify pathways in cancers, and discover gene modules by using information that is available in the WES file. This data is provided by three methods, including SIFT, PolyPhen2, and MutationAssessor [45]. The fourth tool is NetBox, and it can detect driver mutations based on a network. First, a global human interaction network is constructed by this tool. Then, it finds the linker genes between mutated genes for module discovery and identification of candidate drivers [46]. Indeed. the concept and criteria of selecting these four software tools are based on the study in 2015 where the performance metrics of ten methods for prediction of plausible driver genes of BRCA and ovarian OV were compared [19].

#### Feature extraction and feature vector construction

**Fig. 2:**
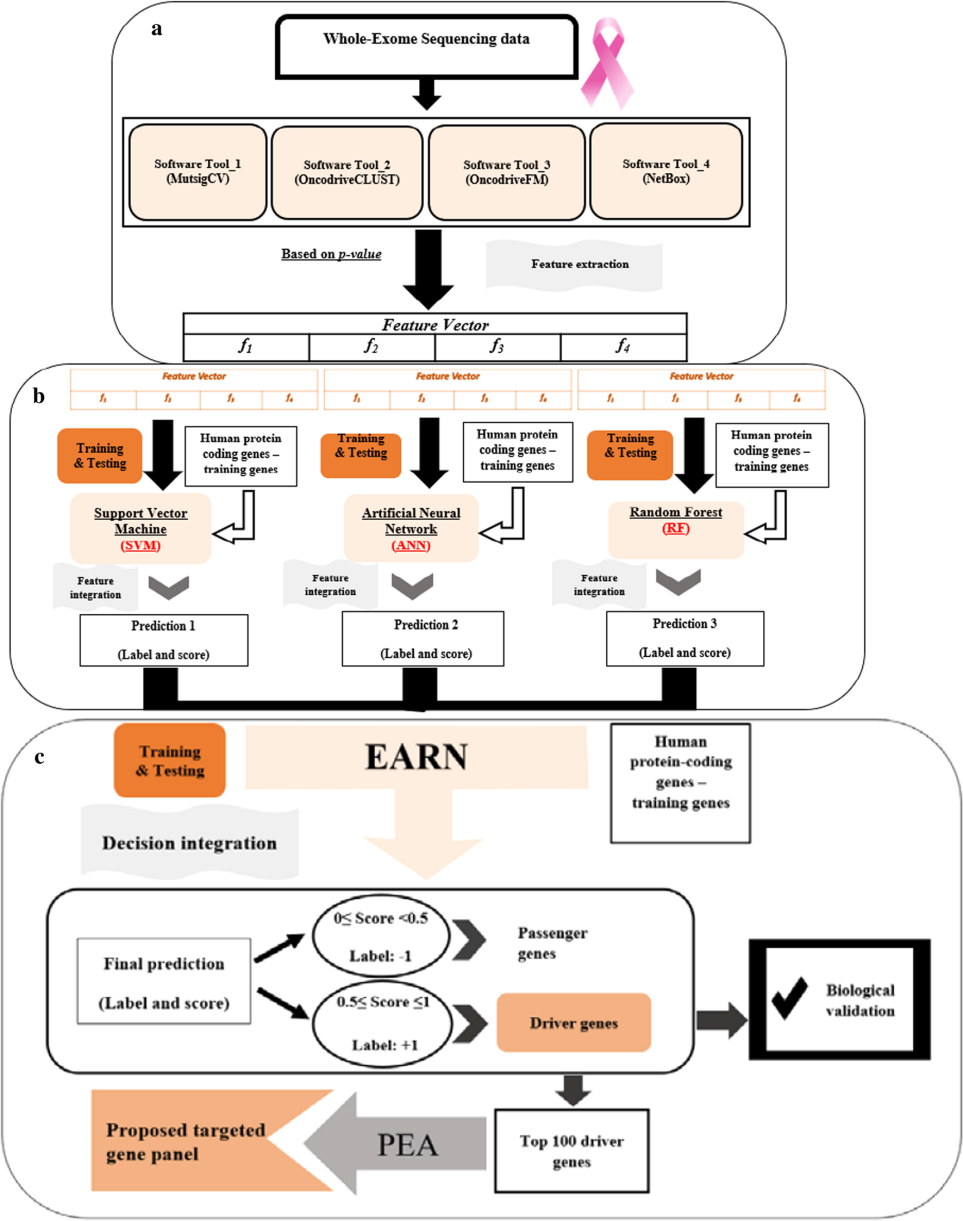
The Workflow for software tools and machine learning methods. **a** Feature extraction and Feature vector construction, **b** feature integration, **c** decision integration

In this step, the four software tools explained above are used for the extraction of features from primary and metastasis mutation data files. After running the tools, all genes are ranked based on *p* value as output data, and each method assigns a number (0 ≤ *p* value ≤ 1) to genes as numerical features. Therefore, a four-dimensional feature vector is constructed for each gene (Fig. 2a). Since the genes with lower *p* value play a more critical role in the development of cancer, we decided to use "1 − *p* value" as the final numerical feature for each gene. With this plan, the genes that are more important in the occurrence of BRCA and MBCA will also get higher feature values. Different and independent logics behind the ranking mechanisms in the exploited tools guarantee enough diversity between inputs of the ensemble system which is an essential property for efficient fusion methods.

### Classifier model selection

Three supervised machine learning methods, including non-linear SVM to learn non-linear functions to separate the classes, ANN, and RF are used as individual classifiers. For selecting these methods, the literature and previous studies were surveyed. We did a comprehensive review regarding fusion systems and the results showed that SVM has been used as a base classifier in many studies or applied as a baseline for comparison between the performance of the different machine learning methods [57–60]. Since in this case, the positive and negative training gene set for the implementation of learning algorithms are highly imbalanced, 40 positive genes versus 2151 negative genes (refer to 2.4), a solution must be found. It has been demonstrated the SVM classifier can be a robust method for generating optimal results with imbalanced positive and negative datasets [61], especially when an Instance-weighted SVM algorithm is used [62]. So, we weighed this algorithm to get better results. On the other hand, RF is an ensemble machine learning method used as one of the individual classifiers. This method can partially solve the problem of the unbalanced positive and negative training set by bootstrap sampling and can also improve performance, i.e., predictive accuracy reached 88.89% using RF for breast cancer risk prediction [63]. The ANN classifier is another machine learning method with long-lasting profound literature. In 1990, Hansen and Salamon integrated multiple neural networks and improved results [64]. This method is also widely used in biology studies and has achieved high performance. In 2017, it was shown that ANN could be used for the diagnosis of lung cancer [65]. Meanwhile, in this study, the positive training set is small, and recent researches have revealed that ANN may improve performance for problems with small training set sizes and give better performance, especially for problems with time-series data category [66]. All of these reasons and criteria led to the selection of these three machine learning methods as base classifiers of the final ensemble system.

### Training and testing

**Fig. 3:**
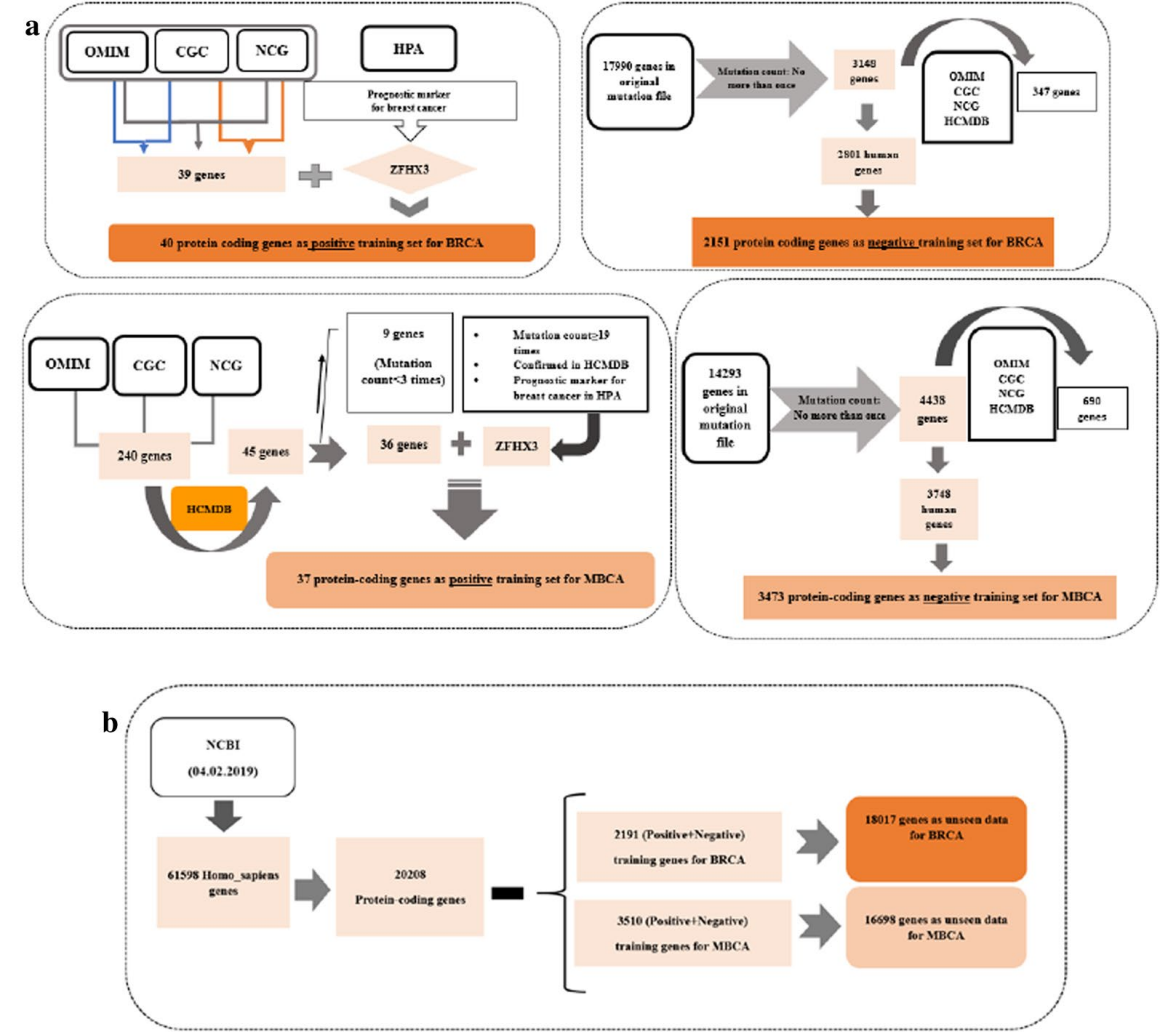
The workflows for the selection of training data and unseen data for BRCA and MBCA. **a** Positive and negative training genes, **b** genome-wide screening

In this study, we train separate models for BRCA and MBCA. Some criteria for the selection of training data sets are described below and visualized in Fig. 3a. For testing the performance of models in terms of evaluation metrics (e.g. recall, precision, etc.), we average over 100 trials. In each trial, 3-fold cross-validation with random shuffles is used to calculate the metrics on all data. Finally, the mean and standard deviation of metrics over 100 trials are obtained. Average outputs for cross-validation of the estimator of each model on testing data based on some metrics, including precision, f1 score, recall, accuracy, and Receiver Operating Characteristic-Area under Curve (ROC-AUC) are presented in “Results” section.

#### Training data set selection

The positive training set of genes for BRCA and MBCA were obtained from searching known genes and mentioned drivers concerning these cancers in several databases, including the OMIM, CGC, NCG, HCMDB, and the Human Protein Atlas (HPA) (https://www.proteinatlas.org/). Also, about selecting negative training gene set, we reviewed a comprehensive list of prior works. Since there is no gold and standard database for a negative set selection, most researchers have used the bootstrap method for resampling, and the negative training genes have been mostly selected randomly. In this study, negative data was selected by counting the occurrence of mutations across all samples in the initial mutation data file, and the genes with the lowest mutation count were used as negative training set [19]. It is crucial to note that in both positive and negative training data, we only accepted protein-coding genes. [see further details for training genes in Additional file 3: Methods and Additional file 4: Table S4–S7].

### Genome-wide screening

For the genome-wide screening, 20,208 homo sapiens genes annotated as protein-coding were downloaded from ftp://ftp.ncbi.nlm.nih.gov/gene/DATA/GENE_INFO/Mammalia/ on February 2019. The proposed ensemble model is applied to 18,017 genes for BRCA and 16,698 genes for MBCA, after excluding positive and negative training sets (Fig. 3b) [see Additional file 4: Table S8–S10].

### Implementation of three machine learning algorithms based on feature integration

After adding features to the system, and training and testing of learning methods including non-linear SVM, ANN, and RF, they are applied to the protein-coding genes as the unseen data. Each of these methods integrates the features extracted from the initial mutation file (refer to “Feature extraction and feature vector construction” section). We use scikit-learn package to implement our algorithms in python [67]. Since this problem is a binary classification of genes based on drivers and passengers, they could label genes based on two indexes − 1 and + 1 (− 1 means passenger genes and + 1 means drivers), and also compute a score for each gene, independently (Fig. 2b).

### Implementation of proposed ensemble machine based on decision integration

Finally, the decision-making strategy for ensemble machine is based on aggregation of the predicted scores obtained from other machines. We call the proposed EC machine learning method EARN (ensemble of ANN, RF, and non-linear SVM). EARN uses the average of the scores of the outputs of the three base classifiers to assign a new score (ranging from 0 to 1) to each gene. The genes with higher prediction scores (scores ≥ 0.5) are labeled as drivers (+ 1) while the other genes will be passengers (− 1). This process has been illustrated in Fig. 2c.

### Biological inferences

At this step, all the driver genes introduced for BRCA and MBCA, as well as top genes predicted by learning machines, are searched in the public databases to determine which genes have been already known related to cancer and which ones are new. Pathway enrichment analysis is also performed using ReactomeFIVIz tool (*FDR* < 0.03) [68–70] to identify the biochemical pathways associated with the candidate genes and examine the biological role of them. It is applied to find biological pathways and patterns related to cancer and other complex diseases.

## Results

This investigation aims to focus on the information achieved from five steps of BRCA and MBCA prognosis and diagnosis. (1) A list of mutated genes ranked by four software tools is presented as the features based on *p* value. (2) Driver genes and passengers predicted by three individual machine learning methods, NLSVM, ANN, RF, and the proposed EC are introduced. (3) Biological validation of predictions is done based on gene set enrichment analysis. (4) Statistical validation of all learning methods is carried out by evaluation metrics. (5) A targeted gene panel for MBCA is proposed by utilizing pathway enrichment analysis (PEA).

### BRCA

The description of the results for BRCA is presented in Additional file 5: Results and Table S11 and S12. However, the comparative results of each algorithm for BRCA and MBCA are illustrated in the next section.

### MBCA

#### Investigation of the diversity of features extracted from the original mutation file

**Fig. 4:**
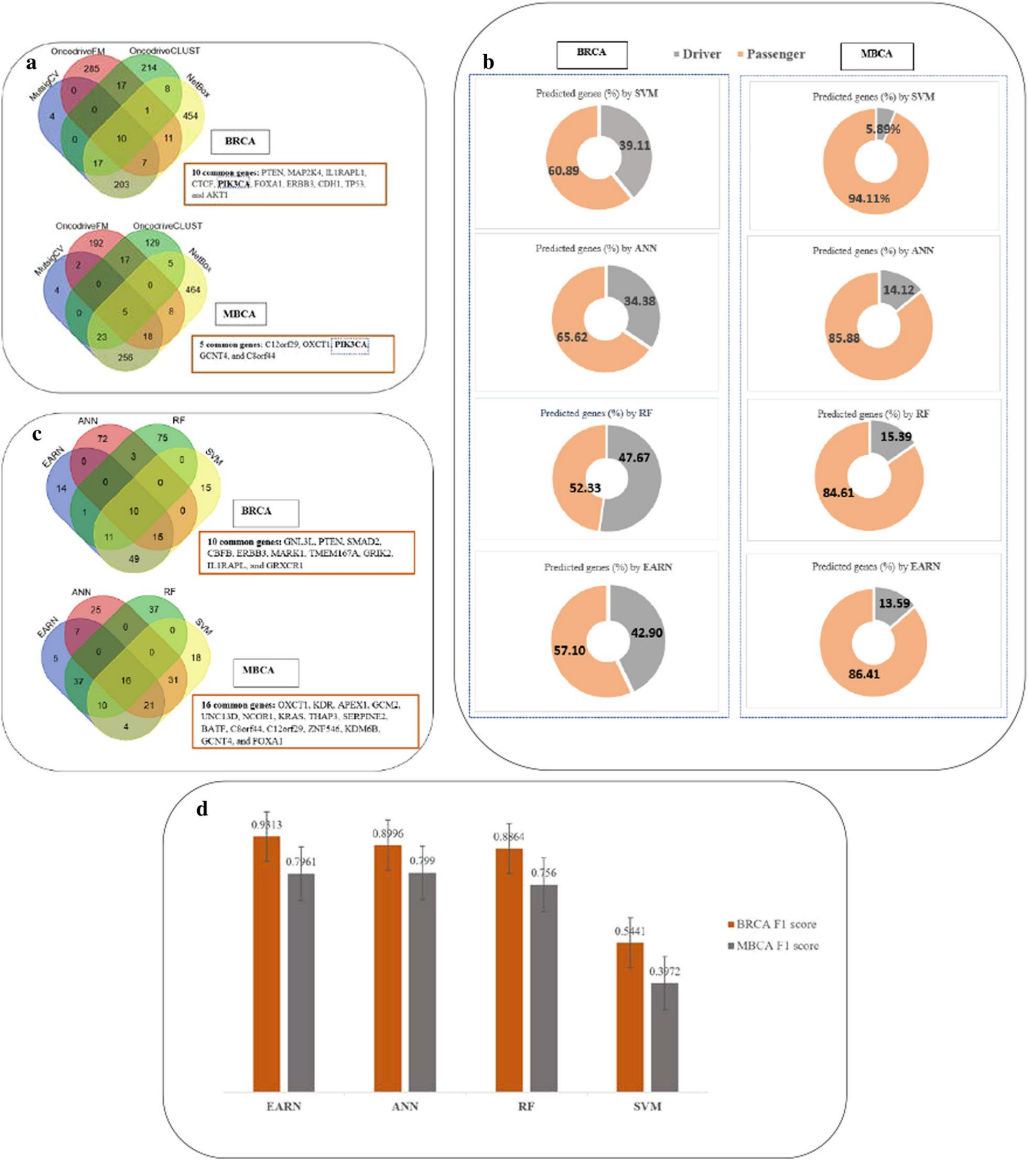
Outputs for BRCA and MBCA. **a** The existence of diversity among features extracted from software tools after setting *p* value ≤ 0.05, **b** frequency of predicted driver and passenger genes using four learning methods, **c** the comparison of driver genes predicted by four methods, **d** the comparison of F1 scores as an evaluation metric of methods

Four software tools are used to extract and rank the list of mutated genes for MBCA as features based on *p* value to be used for the machine learning implementation in the next step. The use of multiple tools for generating features creates an effective diverse committee for better classification. It is known that machine learning method can do better discrimination with higher-dimensional feature vectors and perform the classification with higher accuracy [29]. To illustrate the existence of diversity in features and also for comparison between results of the tools, we plot the GeneVenn diagram [71] by setting *p* value ≤ 0.05 as the threshold. The plotting Venn diagram (*p* value ≤ 0.05) shows that the results of four software tools in the ranking of mutated genes for BRCA and MBCA are varied (Fig. 4a). It means that the extracted features by these tools from the original mutation file are sufficiently diverse and can be applied for machine learning implementation step. The comparison shows that five genes, C12orf29, OXCT1, PIK3CA, GCNT4, and C8orf44, are just common among the outputs. Also, PIK3CA has been selected by all software tools in both cases of BRCA and MBCA [see the outputs of software tools for BRCA and MBCA, and comparison among mutated genes (*p* value ≤ 0.05) extracted by these tools for MBCA in Additional file 6: Table S13–S26].

#### Outputs of three individual classifiers and EARN

The three base classifiers and EARN predicted the labels and scores of 16,698 protein-coding genes for MBCA. The percentage of the predicted driver and passenger genes using the four learning methods for BRCA and MBCA has been shown in Fig. 4b. These findings have been presented in an extra file [see Additional file 7: Table S27–S31].

#### Investigation of top 100 genes predicted by the four machine learning methods

The comparison of the top 100 genes predicted by the four methods using GeneVenn diagram tool shows that 16 genes are predicted by all four machines for MBCA (Fig. 4c). The results of the enrichment of these genes in public databases are considered in Table 2. Other common and unique driver genes predicted by methods are presented in the extra file [see Additional file 8: Table S32–S41]. Also, among the outputs of EARN_100_, BDNF, PRKCG, TH, PRKCD, and PIP5K1B are just predicted by this learning machine in the list of top 100 genes. Among these five genes, BDNF and PRKCG have been already introduced regarding metastatic cancers but the others are new.

**Table 2:** The 16 common genes predicted by all machines in the top 100

Symbol	NSCGMCH (#)	NSCGMBH (#)	PKGECC	PKGEBC
OXCT1^a^	#N/A	#N/A	#N/A	#N/A
KDR^b^	7	2	✓	#N/A
APEX1^a^	#N/A	#N/A	#N/A	#N/A
GCM2^a^	#N/A	#N/A	#N/A	#N/A
UNC13D^a^	#N/A	#N/A	#N/A	#N/A
NCOR1	#N/A	#N/A	✓	✓
KRAS	20	#N/A	✓	✓
THAP3^a^	#N/A	#N/A	#N/A	#N/A
SERPINE2	1	#N/A	#N/A	#N/A
BATF^a^	#N/A	#N/A	#N/A	#N/A
C8orf44^a^	#N/A	#N/A	#N/A	#N/A
C12orf29^a^	#N/A	#N/A	#N/A	#N/A
ZNF546^a^	#N/A	#N/A	#N/A	#N/A
KDM6B	1	#N/A	#N/A	#N/A
GCNT4^a^	#N/A	#N/A	#N/A	#N/A
FOXA1	#N/A	#N/A	✓	✓

The confirmed genes as the known genes related to different primary cancers or primary breast tumors in OMIM, CGC, and NCG databases have been marked in the last two columns

NSCGMCH, number of studies that have cited genes related to different metastatic cancers in the HCMDB; NSCGMBH, number of studies that have cited these genes related to metastatic breast cancer in the HCMDB; PKGECC, predicted known genes by EC associated with different cancers that are confirmed in OMIM, CGC, and NCG; PKGEBC, predicted known genes by EC associated with Breast cancer that are confirmed in OMIM, CGC, and NCG

^a^Ten new genes that have not already been introduced in the databases

^b^KDR is confirmed in HCMDB related to metastatic breast cancer in two studies

#### Biological validation of predictions based on gene set enrichment analysis

The biological analysis of genes predicted by EARN is performed based on two plans; (a) analysis of the results based on all predicted driver genes (labeled as + 1) and (b) analysis of the findings based on the top-scoring genes. To investigate outputs of the EARN for MBCA from a biological point of view based on the label, we analyzed the results concerning the public databases. There is a gene-metastasis association data file (.xls) in the HCMDB that lists 2240 genes related to metastatic cancers based on experiments performed in various studies. 622 genes out of these genes were introduced for metastatic breast cancer specifically. It should be noted that all 37 genes in the positive training gene set have overlap with the gene list of HCMDB in relation to both of different metastatic cancers and metastatic breast cancer. These 37 genes must be excluded to analyze the results. Table 3a, b present the frequency of driver genes enriched in the public databases for MBCA and BRCA.

**Table 3:** The enrichment rate of driver genes predicted by EARN. (a) MBCA, (b) BRCA

All different cancers			Metastatic breast cancer		
HCMDB			HCMDB		
PGEMCH (#)	RGMHP (#)	PGEMCH (%)	PGEMBCH (#)	RGMBHP (#)	PGEMBCH (%)
(a) MBCA					
292	2203	13.25	73^a^	585	12.48

All different cancers			Breast cancer		
OMIM, CGC, and NCG			OMIM, CGC, and NCG		
PKGECC (#)	RKGCPP (#)	PKGECC (%)	PKGEBC (#)	RKGBPP (#)	PKGEBC (%)
(b) BRCA					
1398	2403	58.18	145	201	72.14

PGEMCH, predicted genes by EC associated with different metastatic cancers that are confirmed in HCMDB, RGMHP, remained genes related to different metastatic cancers in the HCMDB after excluding positive training set, PGEMBCH, predicted genes by EC associated with metastatic breast cancer that are confirmed in HCMDB, RGMBHP, remained genes related to metastatic breast cancer in the HCMDB after excluding positive training set, PKGECC, predicted known genes by EC associated with different cancers that are confirmed in OMIM, CGC, and NCG, RKGCPP, remained known genes related to different cancers in the public databases after excluding positive training set, PKGEBC, predicted known genes by EC associated with breast cancer that are confirmed in OMIM, CGC, and NCG, RKGBPP, remained known genes related to breast cancer in the public databases after excluding positive training set

^a^These 73 genes have been also cited in 108 studies of HCMDB [see Additional file 9: S42]

Also, the top 50 genes predicted by all learning methods for MBCA are searched in the list of metastatic cancer-associated genes in the HCMDB. The comparison shows the enrichment score of 24%, 22%, and 16% for RF, ANN, and NLSVM compared to 24% for EARN. Although the value of enrichment in the top 50 is the same for EARN and RF, the number of studies that introduce these enriched genes is 59 for the EARN method compared to 22 for RF. Table 4 presents these genes and also provides more information about them.

**Table 4:** 12 driver genes predicted by EARN50 which are confirmed for metastatic cancers in the HCMDB

Symbol	Prediction score	Rank	PSMM (%) [54]	PSMM (%) [55]	NSCGMCH	NSCGMBH	MCMGM	PKGECC	PKGEBC
APEX1	0.900511991	5	0.50	1.70	1	#N/A	5	#N/A	#N/A
ARID1A	0.895213526	11	2.40	5.10	2	#N/A	24	✓	✓
KDM6B	0.894029187	13	1.40	4.60	1	#N/A	16	#N/A	#N/A
TBX3	0.893837209	14	2.80	5.10	1	#N/A	21	✓	✓
KDR^a^	0.890079401	17	0.90	1.70	7	2	9	✓	#N/A
SERPINE2	0.889205475	19	0.90	0.80	1	#N/A	4	#N/A	#N/A
TBL1XR1	0.871240171	27	0.90	0.80	2	#N/A	4	✓	✓
KRAS	0.868267682	30	1.40	1.70	20	#N/A	7	✓	✓
NOS3	0.861560093	31	2.40	2.10	1	#N/A	12	#N/A	#N/A
RAPGEF3	0.851947423	42	#N/A	2.50	2	#N/A	6	#N/A	#N/A
SELE^a^	0.847865292	49	0.90	1.30	12	1	5	#N/A	#N/A
MME^a^	0.847698297	50	0.90	2.50	9	1	9	#N/A	#N/A

Also, the rank number, score, and mutation count for these genes are provided in the table. The confirmed genes as the known genes related to any primary cancers or primary breast tumors in OMIM, CGC, and NCG databases have been marked in the last two columns

PSMM, Percentage of samples with one or more mutations based on initial mutation file, NSCGMCH, Number of studies that have cited genes related to different metastatic cancers in the HCMDB, NSCGMBH, Number of studies that have cited genes related to metastatic breast cancer in HCMDB, MCMGM, Mutation counts for mutated genes across 450 metastasis tumor samples based on the initial mutation file, PKGECC, Predicted known genes by EC associated with different cancers that are confirmed in OMIM, CGC, and NCG, PKGEBC, Predicted known genes by EC associated with breast cancer that are confirmed in OMIM, CGC, and NCG

^a^These genes have been specifically introduced concerning metastatic breast cancer

Furthermore, 38 genes listed by EARN_50_ have not been introduced in the HCMDB related to any metastatic cancers. So, these genes can be considered as new genes for more investigations [see Additional file 9: Table S43]. For BRCA, the enrichment rate (%) or PPV of the top 50 predictions of the EARN were compared with PPV of the top 50 genes introduced by the four popular software tools, MutSigCV v.1.4, OncodriveCLUST 0.4.1, OncodriveFM, and NetBox 1.0. These tools were also applied in the feature extraction step. All of them have been developed to identify driver genes that are significantly involved in cancer. The comparisons show that EARN achieves a better outcome. PPV for EARN is calculated 52% (26/50). PPVs for MutSigCV, OncodriveCLUST, OncodriveFM, and NetBox are determined 34% (17/50), 20% (10/50), 36% (18/50), and 36% (18/50), respectively. [See the details of these comparisons in Additional file 9, Table S44].

#### Statistical validation of three individual classifiers and EARN based on evaluation measures

For MBCA, a comparison of the metrics based on 3-fold cross-validation on the test data shows that EARN and ANN achieve the best precision with zero FPR. The lower minimum FPR points that no passenger gene is misdiagnosed as drivers. Also, accuracy, F1 score, average precision, and recall for EARN and ANN are better than the others, especially compared with NLSVM. It can be also observed that EARN has the best ROC-AUC (99.24%). Thus, in overall, the proposed EARN outperforms the other three learning methods. For comparison, evaluation metrics of learning methods for MBCA and BRCA are presented in Table 5a, b.

**Table 5:** Validation of four learning methods by some evaluation metrics. (a) MBCA, (b) BRCA

Method name	F1 score	False Positive Rate	Maximum Precision	Average-Precision	Recall	ROC-AUC^a^
(a) MBCA						
EARN	0.7961	0	1	0.8266	0.6701	0.9924
	SD^b^: 0.0264	SD: 0.0	SD: 0.0	SD: 0.0162	SD: 0.0338	SD: 0.0008
RF	0.756	0.0008	0.9069	0.7873	0.6603	0.9418
ANN	0.799	0	1	0.8074	0.6733	0.968
NLSVM	0.3972	0.0154	0.3092	0.5852	0.5885	0.977
(b) BRCA						
EARN	0.9313	0	1	0.9585	0.8749	0.9979
	SD: 0.0117	SD: 0.0	SD: 0.0	SD: 0.0079	SD: 0.0193	SD: 0.0005
RF	0.8864	0.0019	0.9061	0.9171	0.8774	0.9719
ANN	0.8996	0	1	0.9417	0.8225	0.9873
NLSVM	0.5441	0.0279	0.446	0.859	0.8422	0.9926

^a^Receiver Operating Characteristic-Area under Curve

^b^Standard Deviation

In Table 5, we have included standard deviation of the cross-validation classification metrics for EARN to show the confidence interval of the results. It can be observed that the standard deviation is always small, suggesting that the performance metrics are close to the mean value across all experiments. The comparative survey in Table 5 shows when we use a larger mutation dataset (983 tumor samples for BRCA vs. 450 tumor samples for MBCA) for feature extraction, where positive set is larger (40 for BRCA vs. 37 for MBCA), and negative set is smaller (2151 for BRCA vs. 3473 for MBCA), EARN achieves better statistical results. Among all statistical validation metrics, F1 score as a measure of combining the precision and recall has been used to compare performance of the learning methods for both BRCA and MBCA (Fig. 4d).

Also, the performance of classifier pairs, EARN and each base classifier, has been compared using the “K-fold cross-validated paired t-test” procedure [72]. This is a common test for comparing the performance of two models to see if there is a significant difference between the two models and reject the null hypothesis. The comparisons based on *p* value show that EARN performs significantly better than base classifiers [See Additional file 9, Table S45].

### BRCA and MBCA

#### Targeted gene panel discovery for MBCA based on pathway enrichment analysis (PEA)

**Fig. 5:**
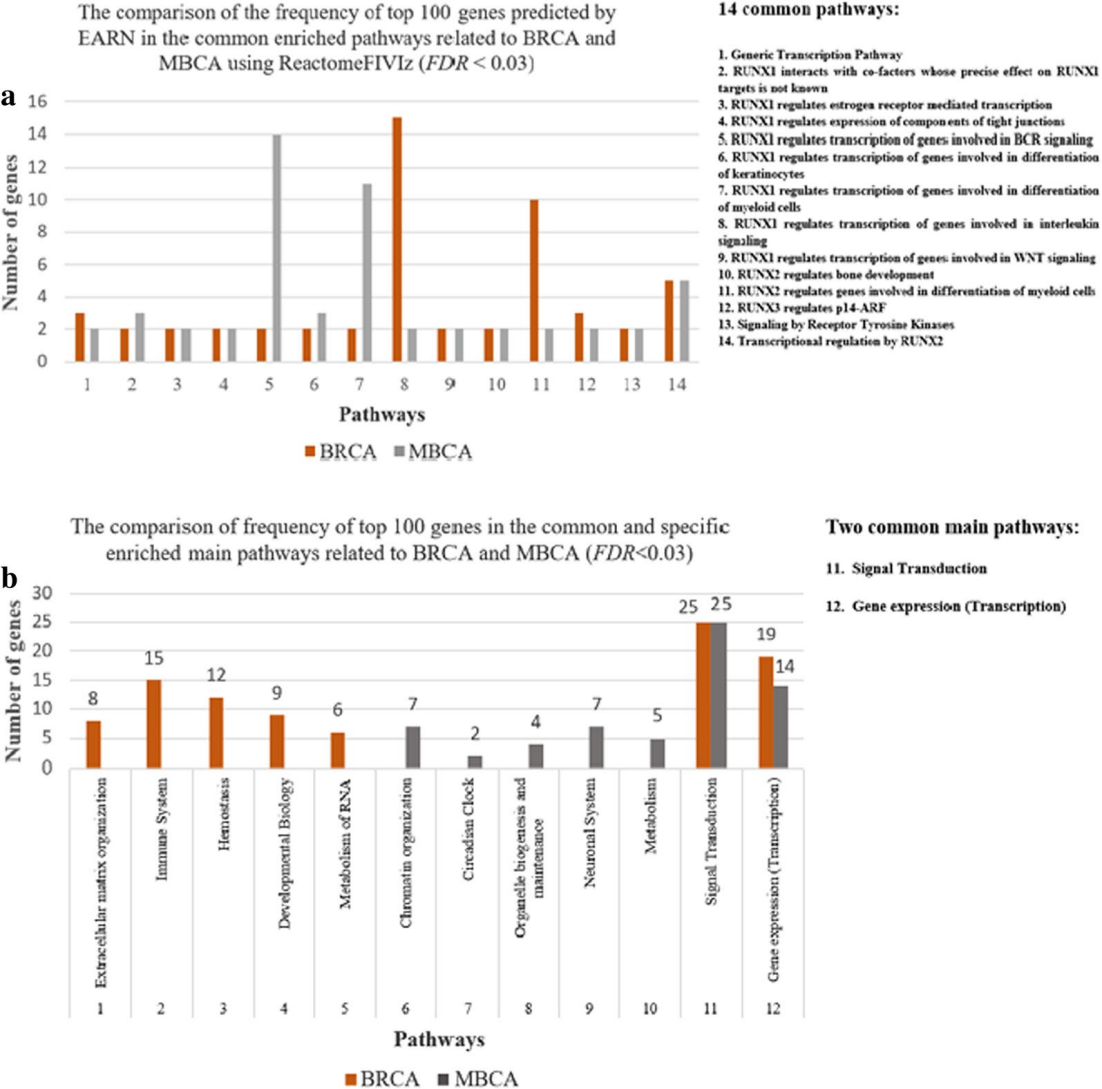
PEA for BRCA and MBCA in top 100 of EARN. **a** The common enriched pathways and the comparison of frequency of top 100 genes predicted by EARN in these pathways. The pathways [1–14] are listed in the guideline box, **b** the common/specific enriched main pathways

In this section, a pathway-based biological analysis is carried out by ReactomeFIVIz tool [68–70]. For EARN_100_, we find 63 (*FDR* < 0.03) such pathways for BRCA and 42 (*FDR* < 0.03) such pathways for MBCA. It is observed that 14 (*FDR* < 0.03) enriched pathways are common among BRCA and MBCA (Fig. 5a), [see these specific and common pathways and the genes involved in each pathway in Additional file 10: Table S46]. These enriched pathways for BRCA are a subset of the other seven main pathways: Extracellular matrix organization, Signal Transduction, Gene expression (Transcription), Immune System, Hemostasis, Developmental Biology, and Metabolism of RNA. Also, the main pathways of MBCA include Gene expression (Transcription), Signal Transduction, Chromatin organization, Circadian Clock, Organelle biogenesis and maintenance, Neuronal System, and Metabolism. The common and specific main pathways (*FDR* < 0.03) of BRCA and MBCA, and the frequency of genes involved in these main pathways are compared in Fig. 5b and Table 6. Given this, it can be found two (*FDR* < 0.03) such common main pathways consist of Signal Transduction and Gene expression (Transcription) for BRCA and MBCA, and 5 (*FDR* < 0.03) such specific main pathways for each of them.

**Table 6:** The common and specific main pathways for BRCA and MBCA

_Number_	_Pathways_	_BRCA_		_MBCA_	
		_Number of genes_	_Name of genes_	_Number of genes_	_Name of genes_
The specific main pathways for BRCA					
1	Extracellular matrix organization	8	DCN, FN1, ICAM1, ITGA4, ITGAM, ITGAV, ITGB3, ITGB5	0	None
2	Immune System	15	FN1, GAB2, ICAM1, IL1RAPL1, IL1RN, IL2RB, ITGAM, ITGAV, ITGB5, JAK1, MSN, POU2F1, PTPN11, SMARCA4, SYK	0	None
3	Hemostasis	12	EGF, FN1, GRB7, ITGA4, ITGAM, ITGAV, ITGB3, PIK3CG, PRKCZ, PTPN11, SERPINA1, SYK	0	None
4	_Developmental Biology_	9	ACVR1B, GAB1, GAB2, GRB7, PTPN11, RELN, SMAD2, SMAD4, VLDLR	0	None
5	_Metabolism of RNA_	6	CPSF1, CPSF3, PCF11, PRPF40A, SF3A1, SF3B1	0	None
The specific main pathways for MBCA					
6	Chromatin organization	0	None	7	TBL1XR1, NCOR1, HDAC3, GPS2, ACTB, KDM6B, PRMT1
7	Circadian Clock	0	None	2	NCOR1, HDAC3
8	_Organelle biogenesis and maintenance_	0	None	4	TBL1XR1, SIRT4, NCOR1, HDAC3
9	Neuronal System	0	None	7	ABAT, KPNA2, PRKCG, CACNA1E, PLCB1, GRIN1, KRAS
10	Metabolism	0	None	5	TBL1XR1, SIN3A, NCOR1, HDAC3, GPS2
The common main pathways for BRCA and MBCA					
11	Signal Transduction	25	ACVR1B, EGF, ERBB3, FLT1, FN1, GAB1, GAB2, GRB7, ITGAV, ITGB3, JAK1, NOTCH4, NR4A1, PARD3, PPARG, PRKCZ, PTEN, PTPN11, RUNX1, SMAD2, SMAD4, SMURF1, SYK, TFDP1, TGFBR2	25	ACTB, AR, BDNF, BUB1B, CBFB, COL4A3, FOXA1, KDR, KPNA2, KRAS, NCOR1, NOS3, PDGFD, PIK3R1, PKN2, PLCB1, PRKCD, PRKCG, PRMT1, PTPRJ, RUNX1, STAG1, STAT1, WAS, YWHAE
12	Gene expression (Transcription)	19	ABL1, CBFB, CPSF1, CPSF3, MED23, NBN, NOTCH4, NR4A1, PCF11, POU2F1, PPARG, PTEN, PTPN11, RUNX1, SMAD2, SMAD4, SMARCA4, SMURF1, TFDP1	14	AR, BDNF, CBFB, GPS2, HDAC3, KLF4, KRAS, NCOR1, PRMT1, RUNX1, SIN3A, STAT1, TBL1XR1, YWHAE

Further investigation in Table 6 shows that 16 genes contribute to five enriched specific main pathways of MBCA. Among them, four genes are involved in more than one main pathway. In particular, NCOR1 and HDAC3 are engaged in four pathways. In three out of five pathways TBL1XR1 is active, and GPS2 gets involved in two pathways. Table 7 introduces 16 genes that are enriched in these five main pathways and provides more information about them.

**Table 7:** The plausible driver genes involved in the proposed main pathways related to MBCA

PPDMB	KGCC	KGBC	CGMC	CGMB	Specific main pathways				
					Chromatin organization	Circadian Clock	Organelle biogenesis and maintenance	Neuronal System	Metabolism
NCOR1	1	1	#N/A	#N/A	✓	✓	✓		✓
HDAC3^a^	#N/A	#N/A	#N/A	#N/A	✓	✓	✓		✓
TBL1XR1	1	1	2	#N/A	✓		✓		✓
SIRT4	1	#N/A	#N/A	#N/A			✓		
ABAT^a^	#N/A	#N/A	#N/A	#N/A				✓	
KRAS	1	1	20	#N/A				✓	
GRIN1^a^	#N/A	#N/A	#N/A	#N/A				✓	
PLCB1^a^	#N/A	#N/A	#N/A	#N/A				✓	
CACNA1E	1	#N/A	#N/A	#N/A				✓	
PRKCG	1	#N/A	1	#N/A				✓	
KPNA2^a^	#N/A	#N/A	#N/A	#N/A				✓	
GPS2	1	1	#N/A	#N/A	✓				✓
SIN3A	1	#N/A	#N/A	#N/A					✓
ACTB	1	#N/A	1	#N/A	✓				
KDM6B	#N/A	#N/A	1	#N/A	✓				
PRMT1	#N/A	#N/A	2	#N/A	✓				

PPDMB, proposed plausible drivers related to metastatic breast cancer; KGCC, known genes related to cancers that are confirmed in OMIM, CGC, and NCG; KGBC, known genes related to breast cancer that are confirmed in OMIM, CGC, and NCG; CGMC, confirmed genes related to different metastatic cancers in HCMDB; CGMB, confirmed genes related to metastatic breast cancer in HCMDB

^a^Five new genes that have not been already introduced in the public databases

**Fig. 6:**
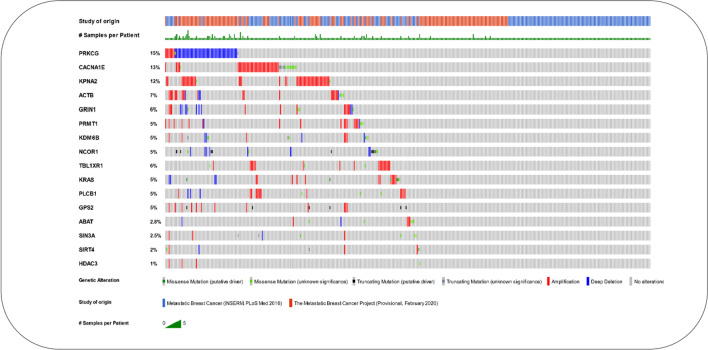
Analysis plot of genomic alterations in 16 proposed genes for MBCA using cBioPortal

**Fig. 7:**
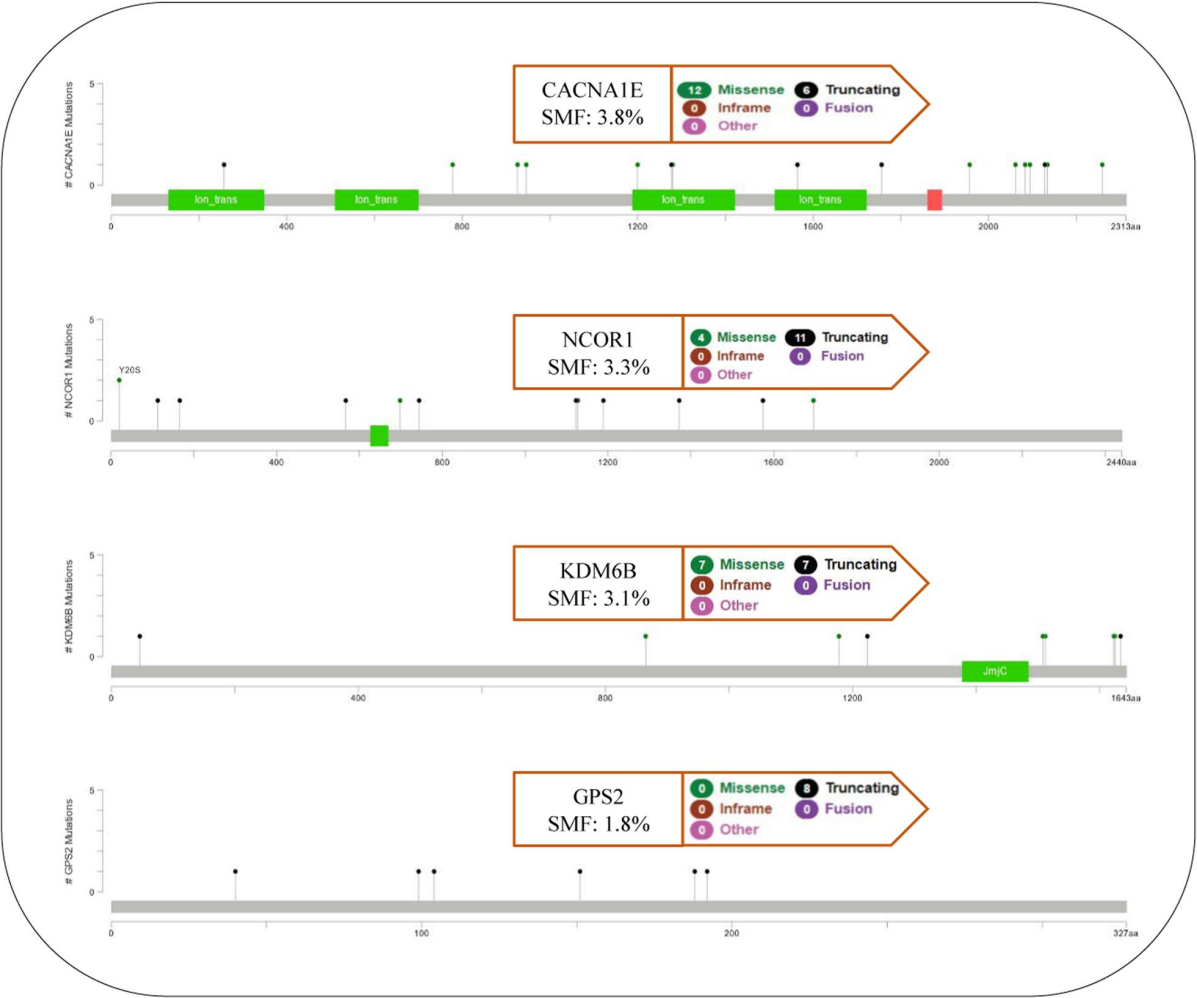
Mutations mapping on a linear protein and its domains using MutationMapper in cBioPortal. SMF (%) and the type of these somatic mutations for four genes are specified in the guideline box

Introducing plausible driver genes confirmed by public databases indicates that EARN has relatively good performance. Thus, other candidate genes in this list have the potential to be considered as a targeted biomarker panel in the case of metastatic breast cancer to examine more in the next molecular and clinical analysis phase. More investigations on these genes can hopefully be helpful in MBCA prognosis and diagnosis. Table 7 shows that five genes, HDAC3, ABAT, GRIN1, PLCB1, and KPNA2 are new and not confirmed in the public databases for cancer prognosis. However, there is some evidence to suggest that these genes play a clinical role in cancer progression. HDAC3 contributes to four pathways alongside NCOR1. The other four genes engage in the Neuronal System pathway. The recent investigations on Basal-like breast cancer (BLBC), the most aggressive subtype of this cancer, have documented the expression of ABAT was considerably decreased in this cancer [73]. Besides, alterations in the expression levels of ABAT have been reported in the promotion of breast cancer [74]. ABAT was also identified as a biomarker for endocrine-responsiveness breast cancer patients [75]. Furthermore, GRIN1 encodes GluN1 subunit of N-methyl-D-aspartate receptor (NMDAR). It has been shown that this subunit in more than 90% of all breast cancer subtypes is uniformly expressed to promote Breast-to-brain metastasis (B2BM) [76]. Recently, the role of HDAC3 in the deregulation of P53 pathway in the aneuploid cancer cell lines has been analyzed [77]. Also, HDAC3 is overexpressed in breast cancer patients. It has been illustrated that breast cancer stem cells, which are resistant to treatment and are responsible for metastasis, are the target of the histone deacetylase (HDAC) inhibitors [78]. PLCB1, Phospholipase C Beta 1, has been also reported that lead to breast cancer development [79]. There is evidence to indicate that KPNA2 is upregulated in breast cancer [80]. On the other, The results of enrichment in cBioPortal show that the above-mentioned 16 genes are altered in 243 (54%) of 450 MBCA samples in two studies performed in 2016 [54] and 2017 [55]. Genomic alterations (Fig. 6) in these genes have been visualized using OncoPrint component [49, 50]. Among them, the highest percentage of somatic mutation frequency (SMF) is observed in CACNA1E, NCOR1, KDM6B, and GPS2. Using the Needle Plot component [49, 50], we visualize SMF and can also map mutations on the linear protein and its domains for these four genes (Fig. 7).

## Discussion

In this work, we proposed an EC machine learning method called EARN, combining three base classifiers to predict and estimate the potential of plausible driver genes in BRCA and MBCA. Also, the architecture of the EARN is such that by availability of the mutation data file, it can be employed for prediction of driver genes of all cancers and in each stage. Leveraged by both feature fusion and decision fusion, the proposed ensemble model made better decisions in comparison with base classifiers, especially in the list of the top genes. Further, we chose EARN which uses the average of the scores of the outputs of the three base classifiers. Meanwhile, the majority voting method as an ensemble strategy among outcomes of individual classifiers was performed. It is a meta-classifier for combining machine learning classifiers via plurality or majority voting among predicted labels by the base classifiers [81]. Next, we compared the results of majority voting with our proposed EARN approach. It can be observed that EARN with averaging strategy achieves better results [See the results of this comparison in Additional file 10, Table S47]. Although EARN uses the simple average operator for aggregating the decisions of the three base learners to predict the driver genes, it could find some new genes in the list of EARN_100_ which were not observed in the top100 of the individual classifiers. It can be rational evidence for using the ensemble systems for gene prioritization. For biological validation of outputs and after the enrichment of EARN_50_ in the public databases, where the ensemble learning method uses most of the power to discriminate and predict, we could obtain the enrichment rate of 52% for BRCA, which outperforms the three individual classifiers. For MBCA, the enrichment of EARN_50_ in the HCMDB resulted in an enrichment rate of 24%, which is better than the two base classifiers, NLSVM and ANN, while being comparable to RF. The results are also analyzed using a statistical test with cross-validation. The evaluation of results showed that EARN performs well, especially for BRCA. In the case of BRCA, the open-access mutation annotation format (.maf) file is large and the mutation data is obtained from more samples (983 BRCA tumor samples vs. 450 MBCA tumor samples). Thus, the proper features could be extracted. Finally, the performance of EARN for ranking human protein-coding genes is improved. Further, to evaluate the possibility of enhancement in the combination of the base classifiers results, we tried StackingCVClassifier, an effective ensemble-learning meta-classifier for stacking [82, 83]. For BRCA, there was no improvement in the results. It could be because the results of the originally proposed ensemble model were good enough. While the metrics such as F1 score (81.31% vs. 79.61%) and recall (69.62 vs. 67.02) were slightly improved for MBCA. Finally, the existence of specific enriched pathways by ReactomeFIVIz (*FDR* < 0.03) for the top genes predicted by EARN for BRCA and MBCA led us to suggest a gene panel regarding metastatic breast cancer. In present study, we faced some limitations to find the appropriate drivers of MBCA. This fact that the original mutation datasets involved in the whole-exome sequencing of the tumor samples of the metastatic breast cancer patients are small. Also, the lack of definitive driver genes confirmed in the public databases for metastatic cancers makes it difficult to select a positive training set. These issues decreased the performance of EARN for MBCA in comparison with BRCA. Further, the result of enriching all predicted genes by EARN for BRCA in the OMIM, CGC, and NCG was encouraging (72.14%, Please refer to Table 3b). But, the result of the enrichment of the predicted genes by EARN for MBCA was not satisfactory (12.48%, see Table 3a). This may be due to the lack of sufficient studies on metastatic cancers, and particularly because of the limited databases regarding metastatic cancers to enrich driver genes.

## Conclusions

Since using computational methods such as ensemble machine learning approaches are less expensive than bio-molecular techniques, it can help to significantly reduce the search space for bio-molecular and medical science researchers in the identification of plausible driver genes to facilitate prognosis and diagnosis of complex diseases. In this work, we mainly focused on the use of genomics data. Meanwhile, the changes of epigenomic, genomic, transcriptional, and proteomic that occur during progression to metastatic encourage us to use multi-omics integration [84]. It has been demonstrated that multi-Omics data integration can improve predictive performance [85] (e.g., it has been applied to predict robust biomarkers of drug efficacy for targeted therapies in triple-negative breast cancer [86]). A direction of future research would be to apply a combination of different levels of data, including genomics, epigenomics, transcriptomics, proteomics, metabolomics, and microbiomics data to optimize the ensemble system for introducing Omics-driven markers. In the end, we strongly emphasize this research needs clinical trials to be validated and to evaluate the potential of the proposed drivers for discrimination between different stages of cancers. By the combination of computational characterization and experimental validation, we can narrow down the list of markers and assist precision oncologists to design compact targeted panels that eliminate the need for whole-genome/exome sequencing.

## Supplementary Information

**Additional file 1: Table S1**. The original mutation files for primary breast tumors

**Additional file 2: Table S2 and S3.** The original mutation files for metastasis breast tumors

**Additional file 3:** Methods: Selection of positive/negative training sets for BRCA and MBCA

**Additional file 4: Table S4–S7.** List of positive/negative gene set for BRCA and MBCA. **Table S8–S10.** Homo_sapiens genes for BRCA and MBCA

**Additional file 5:** Results: Results for BRCA. **Table S11.** Unique driver genes predicted by EARN_100_ for BRCA. **Table S12.** The list of enriched known genes of EARN_50_ in the public databases for BRCA

**Additional file 6: Table S13–S26.** The list of mutated genes extracted by software tools for BRCA and MBCA, and comparison among these genes (*p* value ≤ 0.05) for MBCA

**Additional file 7: Table S27–S31**. The list of driver and passenger genes of four learning machines for MBCA

**Additional file 8: Table S32–S41.** The comparison of drivers predicted by all machine learning methods for MBCA

**Additional file 9: Table S42.** The list of driver genes of EARN for MBCA that have been cited in 108 studies of HCMDB. **Table S43.** The list of novel genes predicted by EARN50 for MBCA. **Table S44.** The comparison of top 50 predictions of EARN and four software tools for BRCA. **Table S45.** The comparison among the performance of classifier pairs based on K-fold cross-validated paired t-test.

**Additional file 10: Table S46.** The common/specific enriched pathways for BRCA and MBCA using ReactomeFIVIz (*FDR* < 0.03). **Table S47.** The comparison of results among averaging model of EARN and majority voting model

## Data Availability

All required data is available in Additional files 1, 2. We have also shared Python source code and other requirements for implementation of the proposed ensemble machine learning algorithm as the protocol via GitHub (https://github.com/lmirsadeghi/EARN/).

## Abbreviations

B2BM: Breast-to-brain metastasis
BLBC: Basal-like breast cancer
BRCA: Primary breast invasive carcinoma
CGC: Cancer Gene Census
DECORATE: Diverse Ensemble Creation by Oppositional Relabeling of Artificial Training Examples
EARN: Ensemble of Artificial Neural Network, Random Forest, and non-linear Support Vector Machine
EC: Ensemble classifier
FPR: False-positive rate
GSEA: Gene set enrichment analysis
HCMDB: Human cancer metastasis database
HPA: Human Protein Atlas
HyDRA: Hybrid Distance-score Rank Aggregation
INDELs: Insertions and deletions
maf: Mutation annotation format
MBCA: Metastatic breast cancer
NCG: Network of Cancer Genes
NGS: Next-generation sequencing
NLSVM: Non-linear Support Vector Machine
NMDAR: N-methyl-d-aspartate receptor
OMIM: Online Mendelian Inheritance in Man
OV: Ovarian
PEA: Pathway enrichment analysis
PPV: Positive predictive value
RF: Random Forest
ROC-AUC: Receiver Operating Characteristic-Area under Curve
SMF: Somatic mutation frequency
TCGA: The Cancer Genome Atlas
WES: Whole-Exome Sequencing
