# Supplementary material for: EARN: an ensemble machine learning algorithm to predict driver genes in metastatic breast cancer
Source: BMC Med Genomics. 2021 May 7;14:122. doi: 10.1186/s12920-021-00974-3 (PMC8105935; doi:10.1186/s12920-021-00974-3)
Supplement: Supplementary file 3 — Additional file 3: Methods: Selection of positive/negative training sets for BRCA and MBCA [file 12920_2021_974_MOESM3_ESM.pdf]

EARN: an ensemble machine learning algorithm to predict driver genes in metastatic breast cancer

Leila Mirsadeghi[1], Reza Haji Hosseini[1]*, Ali Mohammad Banaei-Moghaddam[2], Kaveh Kavousi[3]*

* Correspondence: kkavousi@ut.ac.ir; hosseini@pnu.ac.ir;

[1]Department of Biology, Faculty of Science, Payame Noor University, Tehran, Iran

[2] Laboratory of Genomics and Epigenomics (LGE), Department of Biochemistry, Institute of Biochemistry and Biophysics (IBB), University of Tehran, Tehran, Iran

[3] Laboratory of Complex Biological Systems and Bioinformatics (CBB), Department of Bioinformatics, Institute of Biochemistry and Biophysics (IBB), University of Tehran, Tehran, Iran

Additional file 3: Supplementary Methods

1. Selection of positive training gene set for BRCA

For selecting positive training genes regarding BRCA, three public databases including the OMIM, CGC, and NCG were searched by keyword of breast cancer, and 39 genes were selected. These genes are approved as drivers at least in two databases. Moreover, the ZFHX3 gene has been mentioned as a prognostic marker in breast cancer in the Human Protein Atlas, and by adding it to this list, 40 genes (Fig. 3a) were selected as positive training set [see Additional file 4, Table S4].

2. Selection of negative training gene set for BRCA

Lack of sufficient information about passenger genes makes it difficult to select negative genes. There is no approved list of passenger genes, and one common approach is to select negative genes from pool of remaining genes (those that are not selected as driver) by considering some additional criteria. Similar to other works, here we assume a gene as passenger if (a) the gene mutation rate across all samples is not counted more than once (1/983) in initial mutation file, and (b) it does not overlap with known and candidate driver genes introduced in the OMIM, CGC, NCG, and HCMDB associated with all cancers [15]. By this strategy, 2151 genes (Fig. 3b) were collected as the negative set [see Additional file 4, Table S5].

3. Selection of positive training gene set for MBCA

There are limitations to select the positive training set of genes for metastatic cancers. Data is small, and many studies are underway. To overcome this limitation, we assume a gene can be considered as a driver if it is mentioned in the CGC, OMIM, and NCG as a known gene associated with breast cancer. By this assumption, 240 genes were selected. Among them, 45 genes are confirmed in the HCMDB as genes with significant role in all metastasis tumor progression studies. Finally, 37 genes (Fig. 3c) with mutation count more than three times across all samples in the initial mutation file were considered as a positive set concerned with MBCA [see Additional file 4, Table S6].

4. Selection of negative training gene set for MBCA

For selection of the negative genes for MBCA, there are also the complexities. It was assumed a gene is a passenger concerning MBCA if (a) the mutation count across all samples is no more than once (1/450) in the initial mutation file, and (b) it does not interfere with known and candidate driver genes introduced in the OMIM, CGC, NCG, and HCMDB associated with different cancers [15]. As a result, 3473 genes (Fig. 3d) were introduced as negative by this plan [see Additional file 4, Table S7].
