# Supplementary material for: EARN: an ensemble machine learning algorithm to predict driver genes in metastatic breast cancer
Source: BMC Med Genomics. 2021 May 7;14:122. doi: 10.1186/s12920-021-00974-3 (PMC8105935; doi:10.1186/s12920-021-00974-3)
Supplement: Supplementary file 5 — Additional file 5: Results: Results for BRCA. Table S11. Unique driver genes predicted by EARN100 for BRCA. Table S12. The list of enriched known genes of EARN50 in the public databases for BRCA [file 12920_2021_974_MOESM5_ESM.pdf]

EARN: an ensemble machine learning algorithm to predict driver genes in metastatic breast cancer

Leila Mirsadeghi[1], Reza Haji Hosseini[1]*, Ali Mohammad Banaei-Moghaddam[2], Kaveh Kavousi[3]*

* Correspondence: kkavousi@ut.ac.ir; hosseini@pnu.ac.ir;

[1]Department of Biology, Faculty of Science, Payame Noor University, Tehran, Iran

[2] Laboratory of Genomics and Epigenomics (LGE), Department of Biochemistry, Institute of Biochemistry and Biophysics (IBB), University of Tehran, Tehran, Iran

[3] Laboratory of Complex Biological Systems and Bioinformatics (CBB), Department of Bioinformatics, Institute of Biochemistry and Biophysics (IBB), University of Tehran, Tehran, Iran

Additional file 5: Supplementary Results

Results for BRCA

1. **Investigation of the diversity of features extracted from the original mutation file**

The plotting venn diagram (p-value≤0.05) for BRCA shows that the genes prioritization is diverse and just ten genes are common among the outputs obtained from four software tools regarding BRCA (Fig. 5a). This can be a good indication that the performance of the proposed ensemble model will be appropriate in the next step of implementation of algorithms.

2. **Outputs of three individual classifiers and EARN**

In the case of BRCA and after applying non-linear SVM on 18017 protein-coding genes, 39.11% of predicted genes are labeled with index +1 (Fig. 6a). Similarly, the results are illustrated for the predictions of ANN, and RF in Fig. 6 (b,c). The outputs of EARN for BRCA show 7729 genes (42.90%) out of 18017 genes were identified as drivers and 10288 genes (57.10%) predicted as passengers (Fig. 6d). In comparison, driver genes predicted by EARN are less than RF and more than NLSVM and ANN.

3. **Investigation of top 100 genes predicted by the four machine learning methods**

We compare predicted drivers (top 100 genes) by the four machines using GeneVenn diagram tool (Fig. 7a). 10 genes including GNL3L, PTEN, SMAD2, CBFB, ERBB3, MARK1, TMEM167A, GRIK2, IL1RAPL, and GRXCR1 have been predicted by all the machines. In this list PTEN, SMAD2, CBFB, and ERBB3 were already introduced regarding different cancers in the OMIM, CGC, and NCG databases (PTEN, CBFB, and ERBB3 are also known for breast cancer). The other comparisons are presented in an extra file. The 14 unique driver genes predicted by $EARN_{100}$ have been presented in table S11.

Table S11 14 driver genes predicted only by EARN (EC_{100}) for BRCA

CPSF3	SSX2IP	PARD3
NEK5	SYK	IFI44
SMURF1	DCN	WSCD2
EPS8	ZNF8	RAP1GDS1*
CPSF1	TRMT112	

* In this list, RAP1GDS1 was already introduced in the public databases related to cancers

4. Biological validation of outputs based on gene set enrichment analysis

In the case of BRCA, two plans of the biological inferences of driver genes predicted by EARN are similar to MBCA.

5. The biological inferences of all predicted genes with label +1

For biological analysis based on the label, we calculated the extent to which the driver genes predicted by each method was enriched in the public databases, including the OMIM, CGC, and NCG, associated with different cancers and specifically related to breast cancer. Overall, in these databases, there are 2443 genes as known and candidate genes in occurrence of different primary cancers. We know 40 of these genes are available in the positive training set. In the case of BRCA, the enrichment rate of driver genes predicted by EARN regarding different cancers and breast cancer after excluding 40 positive training genes are 58.18% and 72.14%, respectively.

We also investigated the original mutation file and found that 75.07% of the mutated genes with mutation counts more than 10 across 983 samples were also predicted by EARN as drivers, after excluding positive training genes from the mutated genes list.

6. The biological inferences of predicted top genes

The enrichment of the top 50 genes in three databases shows that the enrichment score for BRCA is 52% using EARN (26/50) compared to 24%, 42%, and 48% (average 38%) related to RF, ANN, and NLSVM, respectively (Table S12).

Table S12 26 confirmed known genes associated with different cancers that are predicted by EARN50 for BRCA

Symbol	Rank number	Prediction score	Mutation count (out of 450 samples)
PTEN*	1	0.9892	34
MED23*	3	0.989	17
ERBB3*	4	0.9889	21
SMAD2	5	0.9886	9
ITGAV	10	0.9874	12
SMARCA4*	11	0.9858	15
MAP3K5	13	0.9851	14
CBFB*	14	0.9842	22
SF3B1*	16	0.984	18
PPARG	18	0.9832	5
NBN	20	0.982	6
CNKSR1	21	0.9804	7
RELN	23	0.9801	31
RUNX1*	29	0.9796	21
ASXL2	30	0.9795	17
TGFBR2*	31	0.9795	5
PTPN11*	35	0.9787	5
ANK3	37	0.9784	38
NOTCH4	38	0.9784	12
NF1*	40	0.978	36
ACVR1B*	41	0.978	12
TLE1	42	0.9779	6
JAK1	47	0.9775	9
PRKCZ	48	0.9774	4
FLT1	49	0.9773	6
TFDP1	50	0.9772	6

* In this list, the 11 genes were already introduced concerning primary breast cancer.

7. Statistical validation of three individual classifiers and EARN based on evaluation measures

For statistical evaluation of the methods, 3-fold cross-validation was done and repeated 100 times to calculate the metrics on test data. For BRCA, comparison of the results of the methods based on statistical validation shows that false-positive rate (FPR), precision or PPV, and average precision for EARN and ANN are better than the other methods. It can be also observed that the recall or sensitivity for EARN and RF is higher than ANN and NLSVM. In comparison, EARN achieves slightly higher accuracy (99.77%) than the others. Also, it can outperform the other methods in F1 score (F-measure) and ROC-AUC.
